# Lower mutant-allele tumor heterogeneity is a biomarker in *FGFR3*-mutant bladder cancer for better prognosis

**DOI:** 10.1186/s12957-020-02084-3

**Published:** 2020-11-26

**Authors:** Yuying Han, Xu Liu, Haihong Ye, Ye Tian, Zhengguo Ji

**Affiliations:** 1 Department of Medical Genetics and Developmental Biology, School of Basic Medical Sciences, Beijing Key Laboratory of Neural Regeneration and Repair, Capital Medical University, Beijing, China; 2 Department of Urology, Beijing Friendship Hospital, Capital Medical University, Beijing, China

**Keywords:** Mutant-allele tumor heterogeneity, *FGFR3*, Prognosis, Bladder cancer

## Abstract

**Background:**

Bladder cancer displays a broad mutational spectrum and intratumor heterogeneity (ITH), which results in difference in molecular phenotypes and resistance to therapies. However, there are currently no clinically available measures to predict patient prognosis using ITH. We aimed to establish a clinically relevant biomarker by using ITH for informing predictive of outcomes.

**Methods:**

We used the Bioconductor R package Maftools to efficiently and comprehensively analyze somatic variants of muscle-invasive bladder cancer (MIBC) from The Cancer Genome Atlas (TCGA). We then used a mutant-allele tumor heterogeneity (MATH) algorithm to measure ITH and explored its correlation with clinical parameters as well as mutational subtypes.

**Results:**

We observed a broad range of somatic mutations in MIBC from TCGA. MATH value was higher for the high-grade group than for the low-grade group (*p* < 0.05). There was a strong correlation between higher MATH value and presence of *TP53* mutations (*p* = 0.008), as well as between lower MATH value and presence of *FGFR3* mutations (*p* = 0.006). Patients with *FGFR3* mutation and low MATH value exhibit longer overall survival time than that of all BLCA patients (*p* = 0.044), which was replicated in another bladder cancer database composed of 109 BLCA patients.

**Conclusion:**

Measures of tumor heterogeneity may be useful biomarkers for identifying patients with bladder cancer. Low MATH value was an independent risk factor that predicted better prognosis for patients with *FGFR3* mutation compared to all BLCA patients.

**Supplementary Information:**

The online version contains supplementary material available at 10.1186/s12957-020-02084-3.

## Introduction

Bladder cancer is one of the most common and highly intratumor heterogeneous malignant tumors of the genitourinary system. Bladder urothelial carcinoma is the most common type, accounting for greater than 90% of all bladder cancers [1]. The majority of bladder cancers occur in men, and there is a wide variation in the incidence and mortality rates worldwide [2]. Based on the degree of tumor invasion, bladder cancers can be categorized as non-muscle-invasive bladder cancer (NMIBC) and muscle-invasive bladder cancer (MIBC). Both NMIBC and MIBC are major sources of morbidity and mortality worldwide. MIBC is associated with greater malignancy, a more diverse mutational spectrum, higher recurrence rate, and worse overall prognosis. Molecular markers have shown potential value in improving diagnostic accuracy of risk stratification of patients [3, 4].

Intratumor heterogeneity (ITH) plays a pivotal role in driving progression and therapeutic resistance in bladder cancer [5]. ITH produces different molecular phenotypes, presenting a significant challenge in the implementation of precision medicine which holds the promise to transform oncology through the use of molecular markers to inform prognosis and guide treatment [6–8].

Based on mutation and gene expression signatures, MIBC can be grouped into five mRNA molecular expression subtypes: luminal-papillary, luminal-infiltrated, luminal, basal-squamous, and neuronal [9, 10]. Molecular subtyping may predict patient survival [9] and response to conventional neoadjuvant chemotherapy [11] and is thus potentially clinically important. *FGFR3* and *TP53* mutations are the most common mutations in MIBC but are mutually exclusive events. Usually, *FGFR3* mutations are accompanied by fewer molecular alterations than are found in *FGFR3* wild-type tumors [9, 12, 13]. *FGFR3*-mutant tumors are significantly correlated with lower-grade bladder cancer, while *TP53*-mutant tumors were found to be strongly associated with a later tumor stage and higher tumor grade. Interestingly, *FGFR3* mutations have been predominantly found in genetically stable bladder cancers with favorable prognoses [12]. Furthermore, anti-FGFR3 therapy slows bladder cancer growth, especially in *FGFR3*-mutant tumors [14]. FGFR3 represents a potentially significant actionable therapeutic target [15], and *FGFR3* can distinguish MIBC subgroup and response to neoadjuvant chemotherapy [16] . However, the heterogeneity of *FGFR3* genotypes within a tumor has not been adequately addressed and may negatively impact therapeutic response [17].

In the present study, we downloaded somatic variants of MIBC in Mutation Annotation Format from the Cancer Genome Atlas (TCGA), and used Maftools [18] to efficiently and comprehensively analyze somatic variants in bladder cancer. We then used a mutant-allele tumor heterogeneity (MATH) algorithm [19] to measure ITH and explore its correlation with clinical parameters. Our integrative analysis reveals the clinical and genetic relevance of ITH in bladder cancer. We identify low MATH value as an independent risk factor that predicts the outcome of *FGFR3*-mutant subtype MIBC patients, which was verified in an independent MIBC database.

## Materials and methods

### Patients and clinical variables

The publicly available mutation data and clinical data used in this study were released by TCGA Data Portal (https: //portal.gdc.cancer.gov) and downloaded from cBioPortal at [http://www.cbioportal.org/datasets]. Another cohort of mutation and clinical data for validation was downloaded from cBioPortal at [http://www.cbioportal.org/datasets].

### Genomic variants and MATH calculation

We downloaded MIBC somatic variants in Mutation Annotation Format (MAF) from the Cancer Genome Atlas (TCGA). We used Maftools, an R Bioconductor package that can be used for integrative analysis of somatic variants, to efficiently and comprehensively analyze somatic variants in bladder cancer.

We calculated the MATH value for each bladder cancer sample according to a previously described protocol [19, 20]. The steps used to determine the MATH value can be summarized as follows: (1) calculate the mutant-allele fraction (MAF) for each locus as the ratio of mutant reads to total reads; (2) obtain the absolute difference of each MAF from the median MAF value and multiply the median of these absolute differences by a constant factor (1.4826) to generate the median absolute deviation (MAD); (3) calculate the MATH value as the ratio of the MAD to the median of the MAFs of the tumor’s mutated genomic loci, presented as a percentage (MATH = 100*MAD/median).

### Statistical analysis

Wilcoxon rank-sum test was used to analyze differences among different variant types. The associations between MATH value and clinical characteristics were evaluated using a Kruskal–Wallis test. Kaplan–Meier survival analyses and log-rank tests were used to estimate the prognoses. All other statistical analyses were performed using IBM SPSS Statistics 25 software. *p* values less than 0.05 were considered statistically significant.

## Results

### Clinical characteristics of patients with bladder cancer

We analyzed genome sequencing data from 412 patients with bladder cancer obtained from the TCGA Bladder Cancer (BLCA) Data Portal. The clinical and pathological characteristics of all patients included in the current analysis are shown in Table 1. High-grade tumors comprised 94.9% of the analysis cohort, whereas low-grade tumors comprised the remaining 5.1%.

**Table 1:** Correlation analysis between clinical characteristics and MATH values in the TCGA-BLCA project

Characteristics	Number of patients	Percent (%)	MATH ± SD	*p* value
Age, median (IQR)	408 (34–90)			*p* = 0.388
≤ 50	25	6.1	41.5 ± 19	
51–74	254	61.3	46.1 ± 16	
≥ 75	133	32.6	46.5 ± 13	
Gender				*p* = 0.527
Male	304	73.8	46.2 ± 16	
Female	108	26.2	45.2 ± 13	
Race				*p* = 0.484
Asian	44	11.2	43.3 ± 19	
Caucasian	327	83.0	46.0 ± 14	
African	23	5.8	45.6 ± 16	
Unkown	18	Unkown	Unkown	
Histologic subtype				*p* = 0.162
Papillary	134	32.8	44.4 ± 18	
Non-papillary	274	67.2	46.7 ± 14	
Unkown	5	Unkown	Unkown	
Stage				*p* = 0.425
Stage I	2	0.5	49.5 ± 50	
Stage II	131	32.0	44.3 ± 16	
Stage III	141	34.4	45.6 ± 15	
Stage IV	136	33.2	47.4 ± 13	
Unkown	2	Unkown	Unkown	
Grade				*p* = 0.024
High-grade	388	94.9	46.2 ± 15	
Low-grade	21	5.1	38.6 ± 18	
Unkown	3	\		
Recurrence				*p* = 0.389
Recur	147	45.1	46.8 ± 15	
Free	179	54.9	45.3 ± 16	
Unkown	86	Unkown	Unkown	

### Comprehensive analysis of somatic variants in bladder cancer

**Fig. 1:**
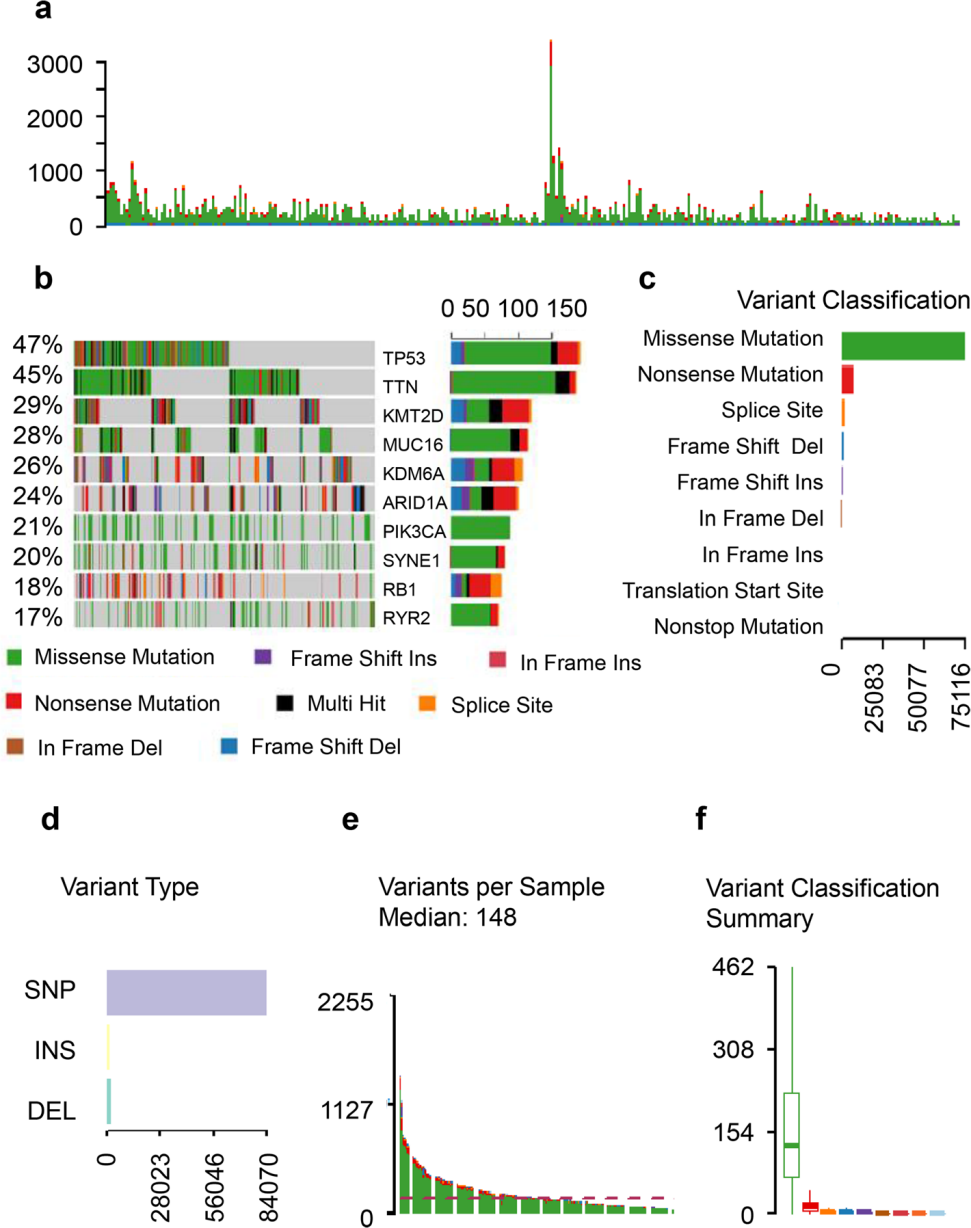
Characteristics of variants identified in the BLCA study cohort. **a** Cumulative frequencies of variants in individual BLCA cases. **b** Oncoplot displaying the somatic landscape of the BLCA cohort. Genes are ordered by their mutation frequency. The side bar plot displays *Q* values estimated by MutSigCV. **c** Bundled bar chart classifying variant types using the variant effect predictor (VEP). **d** Stacked bar chart summarizing the variant types of all cases with substitutions, insertions, and deletions. **e** Histogram showing the cumulative frequency of variants for individual cases. The median of mutation number per sample is 148. **f** Statistical analysis of differences among different variant types (Wilcoxon rank-sum test, *p* < 0.0001)

A total of 87,624 somatic mutations were identified using Maftools. These variants were comprised of 75,116 missense mutations, 7291 nonsense mutations, 1216 insertions, 1,982 deletions, 130 translation start site variants, 1762 splice site variants, and 127 nonstop mutations (Fig. 1a, b, and d). These mutations were further classified using the variant effect predictor (Fig. 1c). The median of total mutation number obtained from each sample was 148 (Fig. 1a, e). There were significant differences among different variant types (Fig. 1f). We identified 50 genes that were mutated in > 10% of samples using Maftools: *TP53* (47%), *TTN* (45%), *MUC16* (28%), *KDM6A* (26%), *SYNE1* (20%), *RB1* (18%), *FGFR3* (14%), *STAG2* (14%), *BIRC6* (11%), *RYR1* (10%), *ADGRV1* (10%), and *AHNAK* (10%) (Fig. 1b and Table S1).

### MATH value characteristics and their relationship with clinical factors

**Fig. 2:**
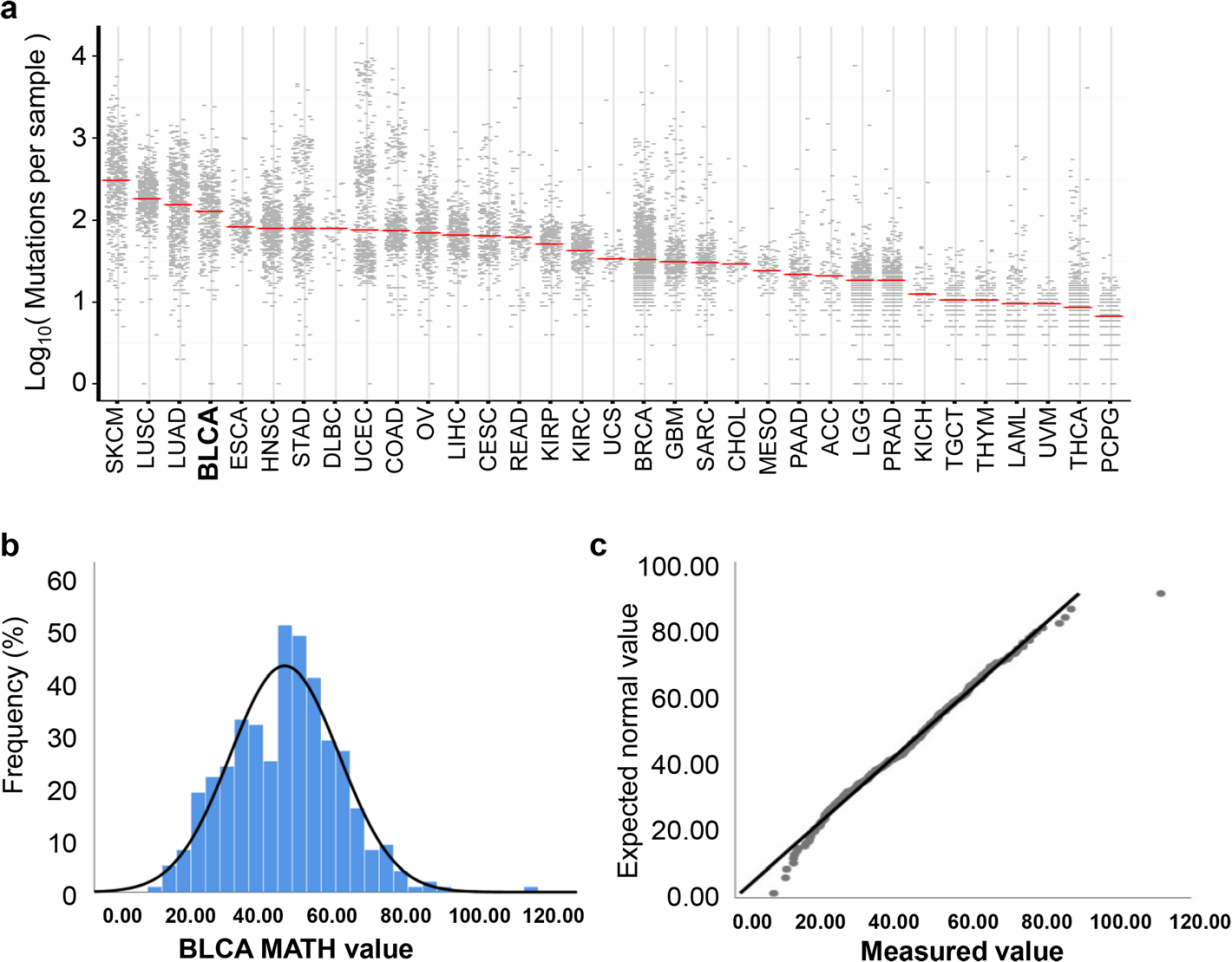
Characteristics of the variants in the BLCA study cohort. **a** Scatter plot comparing the mutational loads of the BLCA cohort with other TCGA cancer cohorts. **b** Distribution of the mutant-allele tumor heterogeneity (MATH) values among bladder cancer patients. **c** Q–Q (quantile–quantile) plot analysis of the distribution of all MATH values

Analysis of the mutation rates of various tumors in TCGA database revealed a high mutation rate for bladder cancer. The median mutational burden for the bladder cancer samples was 148 mutations, which is significantly greater than those of many other cancer types listed in TCGA dataset, ranking fourth in the TCGA tumor category (Fig. 2a). Bladder cancer also displays high ITH during its progression. ITH can be assessed using MATH value. MATH values are calculated from the median absolute deviation (MAD) and the median of its mutant-allele fractions of tumor-specific mutated loci, so the precision of MATH values depends on the sampling of loci and of mutant vs. reference alleles [19]. Thus, MATH value represents a different aspect of tumor biology than the mutation rate. MATH value has been shown to be a simple, quantitative, and generally applicable approach to evaluate the degree of ITH. Here, we calculated MATH values in the TCGA bladder cancer cohort to evaluate the clinical implication of ITH in bladder cancer. The distribution of the MATH values is shown in Fig. 2b. A Q–Q (quantile-quantile) plot (Fig. 2c) and Kolmogorov–Smirnov test (*p* = 0.2) both show that the MATH values from this cohort fit a normal distribution.

**Fig. 3:**
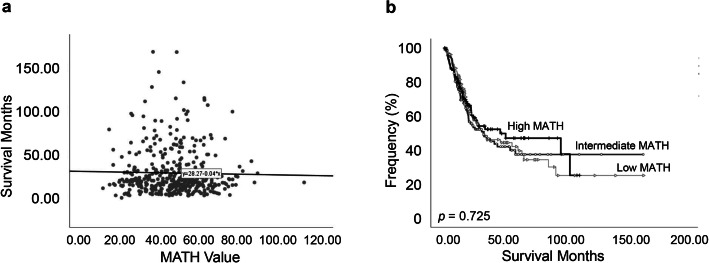
Mutant-allele tumor heterogeneity (MATH) value and its relationship with survival time. **a** Linear dependence relation between MATH value and survival time (*p* = 0.646). **b** Kaplan-Meier survival curves for groups with low, intermediate, and high MATH values. Groups are based on the MATH value cutoffs of the upper and lower tertiles of all MATH values. *p* = 0.725

The upper and lower tertiles of the MATH values were 39.4 and 52.3, respectively. Thus, cases with MATH values less than 39.4 were classified into the “low MATH” group (135 patients; 33.3%) and cases with a MATH value greater than 52.3 were classified into the “high MATH” group (135 patients; 33.3%), while the remainder (MATH value between 39.4 and 52.3) were defined as the “intermediate MATH” group (136 patients; 33.4%). First, we examined the correlation between MATH value and overall survival time in each patient using linear regression and found that MATH value did not exhibit a linear dependence relation with patient’s overall survival time (*p* = 0.646, Fig. 3a). Next, we investigated the prognostic significance of MATH value in the three predefined MATH groups described above. We find that MATH value was not an independent predictor of overall survival time in the entire bladder cancer cohort (*p* = 0.725, Fig. 3b).

**Fig. 4:**
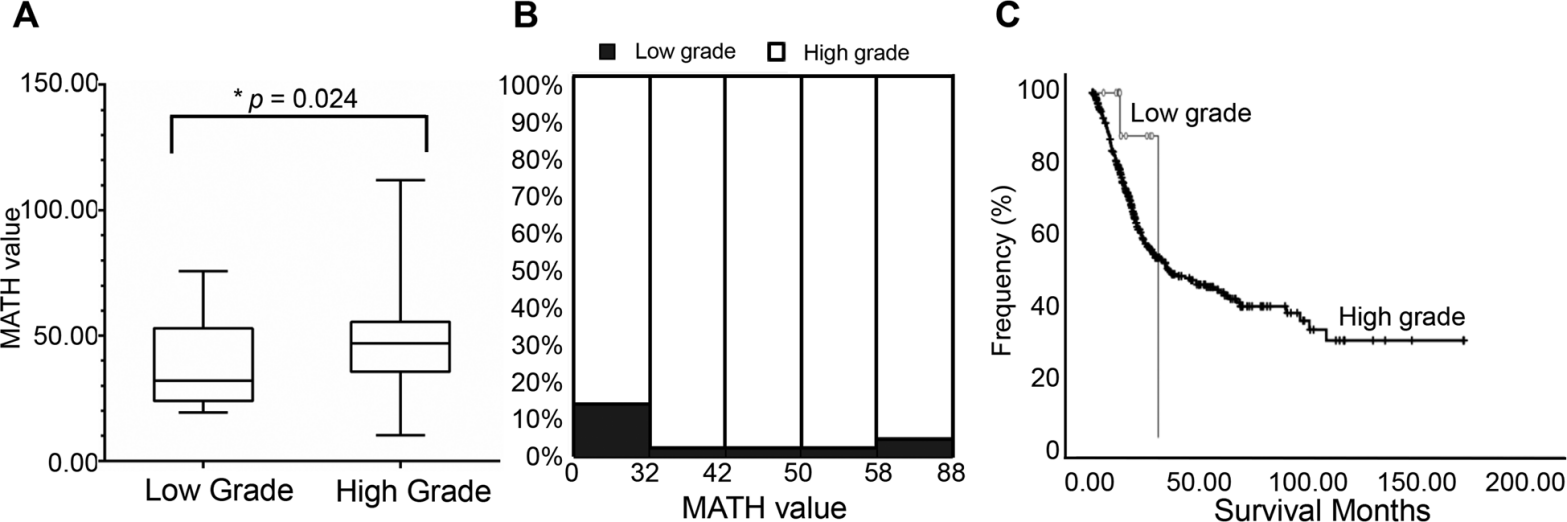
Mutant-allele tumor heterogeneity (MATH) value and its relationship with clinical grade. **a** MATH values for the original low-grade and high-grade tumors (*p* = 0.024). **b** MATH value and its relationship with clinical grade. Each panel represents the relationship between clinical grade and MATH values. Within each panel, the horizontal axis represents the range of tumor MATH values divided into five groups comprised of approximately equal numbers of tumors. **c** Kaplan–Meier survival curves for the low-grade and high-grade groups (*p* = 0.116)

Because the MATH value grouped by grade, stage, or race did not conform to normal distribution, rank sum test was used to test the difference among them. Using rank sum test of variance, the MATH value was found to be significantly related to tumor grade (*p* = 0.024), but not to race (*p* = 0.484) or stage (*p* = 0.425) (Table 1). Using one-way ANOVA, the MATH value was not found to be related to recurrence (*p* = 0.389) or tumor histologic subtype (*p* = 0.162) (Table 1). Figure 4a shows the range of calculated MATH values for tumors initially labeled low-grade and high-grade. Higher MATH values were specifically related to a high tumor grade. MATH value was higher in the high-grade group than in the low-grade group (*p* < 0.05), which suggests that MATH value may be useful in differentiating such patients. We next analyzed the percentage of low- and high-grade cases with different ranges of tumor MATH values. The high-grade cases tended to show a significantly increased level of MATH value than that of the low-grade cases (Fig. 4b). We then explored the correlation of MATH value and survival in low-grade and high grade subgroups. Interestingly, MATH value was not an independent prognostic factor in the low- and high-grade patients (Fig. 4c, *p* = 0.116). Taken together, we found that MATH value was not an independent risk factor for survival time, but a high MATH value was related to high-grade BLCA.

### Low MATH value was an independent favorable prognostic biomarker in *FGFR3*-mutant patients

**Fig. 5:**
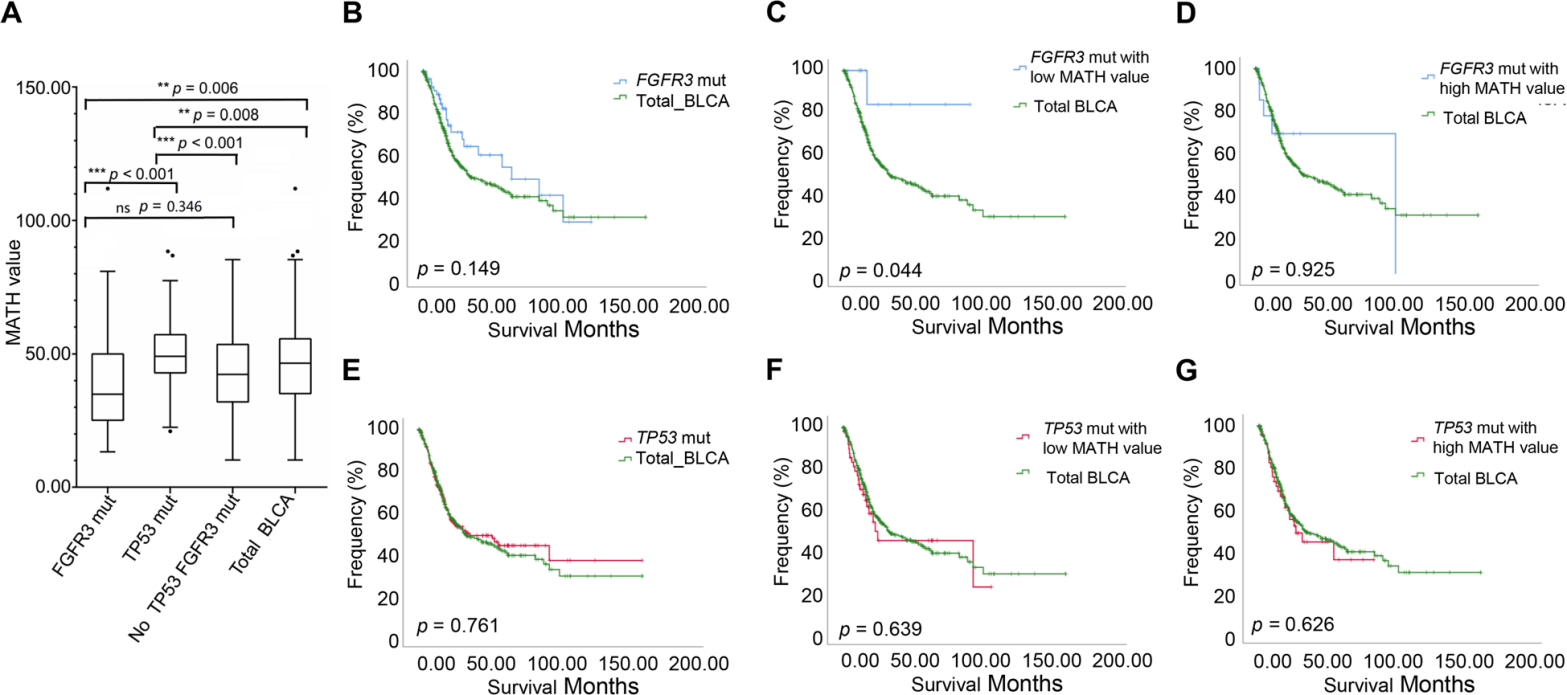
Prognostic evaluation of MATH value in *TP53* and *FGFR3* mutations patients. **a** MATH values for *TP53*-mutant, *FGFR3*-mutant, and no mutation with *TP53* and *FGFR3*, Kruskal-Wallis test (*p* < 0.05). **b–g** Kaplan–Meier survival analysis comparing patients in specific subgroups to all patients in the BLCA cohort from TCGA

Although MATH value was not related to the overall mutation rate, we hypothesized that different driver gene mutations may direct different routes of tumor evolution, resulting in variable intratumor heterogeneity. To test this, we analyzed the mutual exclusivity and co-occurrence of the top 50 mutated genes in BLCA based on cBioPortal [21, 22] (Table 2). We observed mutually exclusive variants in *FGFR3* vs. *TP53* (*q* < 0.003) and *FGFR3* vs. *RB1* (*q* < 0.02). Similar analyses identified co-occurrence of variants in *TP53* vs. *RB1*, *RYR1* vs. *AHNAK*, *BIRC6* vs. *ADGRV1*, *SYNE1* vs. *AHNAK*, *FGFR3* vs. *STAG2*, *KDM6A* vs. *STAG2*, *MUC16* vs. *BIRC6*, and *TTN* vs. *MUC16* (*q* < 0.005). In the BLCA cohort, *TP53* was mutated in > 47% of the samples, and *FGFR3* was mutated in > 14% of the samples. *FGFR3* and *TP53* mutation are potential survival prediction biomarkers of MIBC. The known role of the p53, as a guardian of the genome stability [23], supports the general hypothesis that *TP53* mutations lead to increased ITH. Thus, we first aimed to validate the hypothesis that *TP53* mutations, rather than *FGFR3* mutations, was associated with greater ITH in BLCA. Based on the presence of somatic mutations in *TP53* and *FGFR3*, we divided the BLCA cohort into three groups: *TP53*-mutant, *FGFR3*-mutant, and no *TP53* and *FGFR3*-mutant. Using MATH value as a measure of ITH, we examined the relationship of heterogeneity between the above three groups. Consistent with our hypothesis, *TP53* mutations were specifically associated with higher MATH values. MATH values were higher in the BLCA cases with *TP53* mutations than in those with *FGFR3*-mutant (Fig. 5a, *p* < 0.001) or no *TP53*- and *FGFR3*-mutant (Fig. 5a, *p* < 0.001) group. MATH values were lowest in BLCA cases with *FGFR3* mutations (Fig. 5a).

**Table 2:** Mutual exclusivity and co-occurrence of the top 50 mutated genes in BLCA

A	B	Neither	A not B	B not A	Both	Log_2_ odds ratio	*p* value	*q* value	Tendency
*TP53*	*FGFR3*	169	185	45	13	− 1.922	< 0.001	0.003	Mutual exclusivity
*RB1*	*FGFR3*	284	70	56	2	− 2.787	< 0.001	0.02	Mutual exclusivity
*TP53*	*RB1*	199	141	15	57	2.423	< 0.001	< 0.001	Co-occurrence
*RYR1*	*AHNAK*	337	33	25	17	2.796	< 0.001	< 0.001	Co-occurrence
*BIRC6*	*ADGRV1*	333	34	28	17	2.572	< 0.001	0.001	Co-occurrence
*SYNE1*	*AHNAK*	307	63	21	21	2.285	< 0.001	0.002	Co-occurrence
*FGFR3*	*STAG2*	317	38	37	20	2.173	< 0.001	0.002	Co-occurrence
*KDM6A*	*STAG2*	277	78	28	29	1.879	< 0.001	0.002	Co-occurrence
*MUC16*	*BIRC6*	272	89	23	28	1.896	< 0.001	0.003	Co-occurrence
*TTN*	*MUC16*	174	121	43	74	1.307	< 0.001	0.005	Co-occurrence

Next, we investigated the prognostic significance of MATH value in the *TP53*-mutant and the *FGFR3*-mutant groups. For *FGFR3*-mutant patients, we observed a trend of increased survival time compared to all patients in the BLCA cohort, although the change was not significant (Fig. 5b, *p* = 0.149). We then divided the *TP53*- and *FGFR3*-mutant groups into low and high MATH value subgroups, respectively, using quartile MATH values as cut-offs. We found that low MATH value was a significant predictor of better survival in the *FGFR3*-mutant group (Fig. 5c, *p* < 0.05). On the contrary, we found no significant difference of overall survival in *FGFR3*-mutant with high MATH value compared to all patients in the BLCA cohort (Fig. 5d, *p* = 0.925), which indicated that the better survival of patients was specifically correlated with patients carrying *FGFR3* mutation with lower MATH value. At the same time, we compared the overall survival between *TP53*-mutant and all patients in the BLCA cohort. There was no significant difference between them (Fig. 5e, *p* = 0.761). Moreover, there was no significant difference in overall survival comparing *TP53*-mutant with either low MATH value or high value to all patients in the BLCA cohort, respectively (Fig. 5f, *p* = 0.639; Fig. 5g, *p* = 0.626;), which indicated that MATH value was not a biomarker for prognosis in *TP53*-mutant patients. Taken together, we found that low MATH value was an independent favorable prognostic biomarker in *FGFR3*-mutant patients.

### Validation of low MATH value as a prognostic biomarker in *FGFR3-*mutant patients in an independent BLCA cohort

**Fig. 6:**
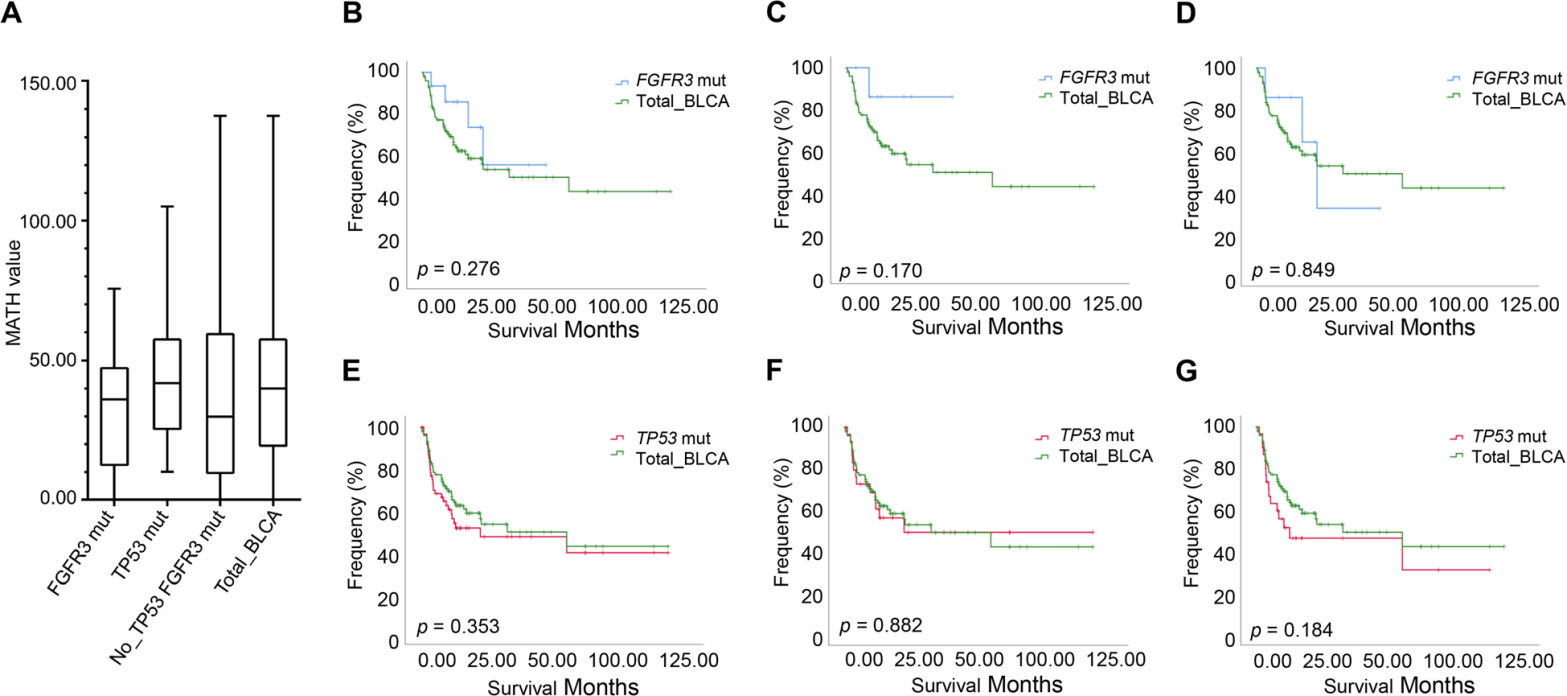
Validation and prognostic evaluation of MATH value in *TP53* and *FGFR3* mutation patients in an independent BLCA cohort. **a** MATH values for *TP53*-mutant, *FGFR3*-mutant, and no mutation with *TP53* and *FGFR3*, Kruskal–Wallis test (*p* > 0.05). **b–g** Kaplan–Meier survival analysis comparing patients in specific subgroups to all patients in an independent BLCA cohort

To further investigate whether low MATH value in *FGFR3*-mutant patients is widely applicable to predict better overall survival in BLCA, we selected another BLCA cohort which was composed of 109 bladder cancer patients published in 2015 [24]. We download all mutation and clinical data from cBioPortal and reassessed its prognostic value. First, to verify that *FGFR3* mutations, rather than *TP53* mutations, were associated with lower ITH in BLCA, we divided the 109 samples [24] into three groups: *TP53*-mutant, *FGFR3*-mutant, and no *TP53* and *FGFR3*-mutant. Using MATH as a measure of ITH, we examined the relationship of heterogeneity between the above three groups. Although there was no significant difference between them (*p* > 0.05), probably due to the small sample size in this cohort, the overall trend of the median MATH values showed that MATH values were greater in BLCA patients with *TP53* mutations than in those with *FGFR3* mutations (Fig. 6a, *p* = 0.074) and MATH values were lowest in BLCA cases with *FGFR3* mutations (Fig. 6a), consistent with our findings in the TCGA BLCA cohort (Fig. 5a).

Next, we investigated the prognostic significance of MATH value in the *FGFR3*-mutant and *TP53*-mutant groups. In all *FGFR3*-mutant patients, we observed a trend of increased survival time compared to all patients in the whole cohort (Fig. 6,b *p* = 0.279). We divided *FGFR3*-mutant patients and *TP53*-mutant patients into low MATH value subgroup and high MATH value subgroup, respectively. The prognosis trend of *FGFR3*-mutant patients with low MATH value was better than that of the patients in the whole cohort, although the difference was not statistically significant, probably due to the limited number of patients in this subgroup (Fig. 6c, *p* = 0.170). We found no significant difference of overall survival in any other subgroups (Fig. 6e–g), indicating that MATH value was not a biomarker for prognosis in the *TP53*-mutant patients. Taken together, we verified that low MATH value was an independent favorable prognostic biomarker in *FGFR3*-mutant patients in another bladder cancer cohort.

## Discussion

Bladder cancer, including both NMIBC and MIBC, is a major source of morbidity and mortality worldwide. MIBCs have a more diverse mutation spectrum [25, 26] and display high overall mutation rates similar to those of non-small cell lung cancers and melanoma [27]. MIBCs are also highly recurrent cancers worldwide [28].

Similar to most solid tumors, BLCA is spatially heterogeneous. ITH is the result of temporal acquisition of mutations and corresponding tumor evolution. ITH is observed at both the genomic and the transcriptomic levels. ITH plays a pivotal role in driving bladder cancer progression and resistance to therapies. High genetic heterogeneity is thought to contribute to risk of poor survival in patients with bladder cancers. MATH value is a novel approach to quantify ITH. In several previous studies, it was reported as a prognostic biomarker for patients with head and neck squamous cell carcinoma [19, 20]. Any personalized prognostic indicator or tumor treatment requires a test to distinguish patients who will benefit from those who will not [29]. However, differences in ITH in MIBC evaluated using MATH value have not been thoroughly explored at the genomic level. Our study represents the first attempt to describe the prognostic value of MATH value.

Precision medicine is transforming oncology through the use of molecular markers to guide treatment and inform prognosis [6]. MIBC has a more diverse mutant spectrum (as shown in Table S1). Different driver gene mutations may direct different routes of tumor evolution which may affect the prognosis of bladder cancer patients. MATH value was not related to the overall mutation rate and depended on the mutation frequency of multiple mutated genes. Therefore, the MATH value was not the only factor affecting the prognosis of bladder cancer patients, and the mutation of the driver gene needs to be considered. *FGFR3* and *TP53* are the most common mutations in MIBC, but are mutually exclusive events. Therefore, we combined *FGFR3* and *TP53* mutations and MATH value to analyze the prognosis of bladder cancer patients. *FGFR3* mutation is usually accompanied by fewer molecular alterations than wild-type *FGFR3* [12, 13]. The p53 protein functions as a guardian of the genome stability, which supports the hypothesis that mutations in *TP53* result in increased ITH. We examined the relationship between heterogeneity (using MATH value) with *FGFR3* and/or *TP53* mutations and found that *FGFR3* mutations were associated with lower ITH. The administration of neoadjuvant chemotherapy preceding radical cystectomy improves overall survival of patients with MIBC [30], and *FGFR3* mutations in MIBCs are potential predictive biomarkers of the response to neoadjuvant [16]. In most primary invasive bladder cancers, FGFR3 status can guide the selection of FGFR targeted therapy [31]. Our study showed that *FGFR3*-mutatant cases have the characteristics of low MATH value, which provides a valuable reference standard for neoadjuvant chemotherapy. We also assessed the prognostic value of MATH with *TP53* or *FGFR3* mutations and found that a low MATH value was an independent risk factor that predicted better survival in the *FGFR3*-mutant patients. This may be due to better sensitivity to cisplatin and gemcitabine treatment in this subgroup of patients, which requires further clinical and genetic studies to verify.

Given the nature of available BLCA cohorts, several potential limitations exist in our current analysis. Although properly powered, the bladder cancer cohorts used in our study lacked several clinic pathological parameters. The follow-up data of patients are not uniform, so there are some restrictions on the clinical correlation analysis. Moreover, the information on the pre- and postoperative treatment is not available in the majority of the patients with bladder cancer, and thus, these impacts on overall survival analysis could not be fully considered. Furthermore, the majority of BLCA patients were of the Caucasian race, so the predictive significance for other populations is questionable. Further studies using clinical samples from other racial groups with complete clinical data and follow-up data are necessary, and further verification together with multiple algorithms are needed.

MATH value provides a quantifiable estimation of ITH and is less affected by the limitation of somatic mutation number and sequencing depth in the bladder cancer cohorts. However, the ITH represented by the MATH value might be confounded by the load of somatic copy number alterations, which should not be ignored. Using MATH value to quantify tumor heterogeneity is valuable, but the standards are different for each somatic caller in different databases, so the results from different databases are different. Therefore, the use of MATH value to quantify tumor heterogeneity must unify the adoption standard of somatic mutation.

In our study, we found that MATH value as a measure of ITH may be useful biomarkers for patients with bladder cancer. Here, we show that patients with *FGFR3* mutations and low MATH value tend to exhibit favorable prognosis. ITH at the genome level should be taken into account to guide management of bladder cancer patients.

## Conclusions

Bladder cancer is a malignant tumor with extensive mutant spectrum and high tumor heterogeneity. MATH value is effective in evaluating tumor heterogeneity. Measures of tumor heterogeneity may be useful biomarkers for identifying patients with bladder cancer. Low MATH value was an independent risk factor that predicted better prognosis for patients with *FGFR3* mutation compared to all BLCA patients.

## Supplementary Information

**Additional file 1:Table S1** The top 50 mutated genes in BLCA.

## Data Availability

The datasets used and/or analyzed during the current study are available from the corresponding author on reasonable request.

## Abbreviations

BLCA: Bladder cancer
ITH: Intratumor heterogeneity
MAF: Mutant-allele fraction
MATH: Mutant-allele tumor heterogeneity
MIBC: Muscle-invasive bladder cancer
NMIBC: Non-muscle-invasive bladder cancer
TCGA: The Cancer Genome Atlas
